# Topical antifungal keratitis therapeutic potential of *Clitoria ternatea* Linn. flower extract: phytochemical profiling, *in silico* modelling, and *in vitro* biological activity assessment

**DOI:** 10.3389/fmicb.2024.1343988

**Published:** 2024-01-24

**Authors:** Poomany Arul Soundara Rajan Yolin Angel, Palanisamy Jeyakumar, Arul Raj Jasmin Suriya, Aliyas Sheena, Ponmurugan Karuppiah, Govindasami Periyasami, Antony Stalin, Kasi Murugan

**Affiliations:** ^1^Biofilm and Bioprocess Laboratory, Department of Biotechnology, Manonmaniam Sundaranar University, Tirunelveli, Tamil Nadu, India; ^2^Department of Botany and Microbiology, College of Science, King Saud University, Riyadh, Saudi Arabia; ^3^Department of Chemistry, College of Science, King Saud University, Riyadh, Saudi Arabia; ^4^Institute of Fundamental and Frontier Sciences, University of Electronic Science and Technology of China, Chengdu, China

**Keywords:** Fungal keratitis, *C. ternatea*, phytocompounds, pharmacokinetics, topical drug

## Abstract

**Introduction:**

Fungal keratitis (FK) poses a severe threat to vision, potentially leading to blindness if not promptly addressed. *Clitoria ternatea* flower extracts have a history of use in Ayurvedic and Indian traditional medicines, particularly for treating eye ailments. This study investigates the antifungal and antibiofilm effects of *Clitoria ternatea* flower extracts on the FK clinical isolate *Coniochaeta hoffmannii*. Structural details and key compound identification were analysed through FTIR and GC-MS.

**Methods:**

The minimum inhibitory concentration (MIC) and minimum fungicidal concentration (MFC) of *Clitoria ternatea* flower extracts were determined using broth dilution and well plate techniques. Biofilm inhibitory activity was assessed through microscopic evaluation, while anti-irritant and cytotoxic properties were evaluated using CAE-EI and MTT assays. Through GC-MS and FT-IR analysis the compounds dissolved in the extract and their functional group were studied, and their toxicity screening and pharmacokinetic prediction were conducted *in silico*. Subsequently, compounds with high corneal permeability were further identified, and molecular docking and simulation studies at 150 ns were used to investigate their interactions with fungal virulence factors and human inflammatory proteins.

**Results and Discussion:**

At a concentration of 250 µg/mL, the *Clitoria ternatea* flower extract displayed effective biofilm inhibition. MIC and MFC values were determined as 500 and 1000 µg/mL, respectively. CAE-EI and MTT assays indicated no significant irritant and cytotoxic effects up to a concentration of 3 mg/mL. Compounds like 9,9-dimethoxybicyclo[3.3.1]nonane-2,4-dione showed high corneal permeability with strong and stable interactions with fungal virulence cellobiose dehydrogenase, endo β 1,4 xylanase, and glucanase, as well as corneal inflammation-associated human TNF-α and Interleukin IL-1b protein targets. The findings indicate that extracts from *C. ternatea* flowers could be formulated for an effective and safe alternative for developing new topical FK therapeutics.

## Graphical abstract

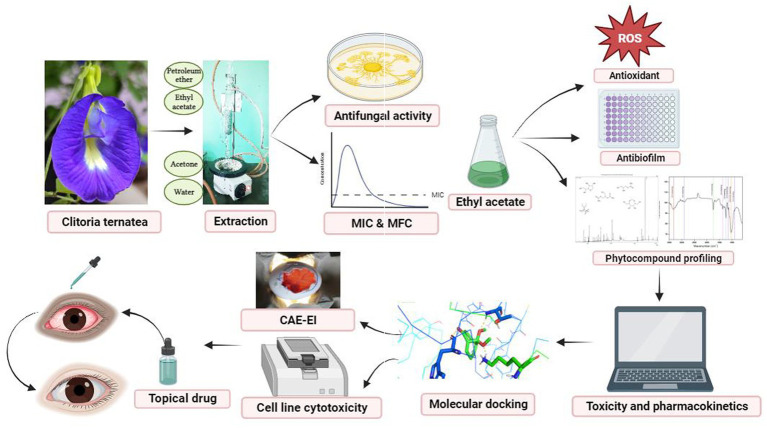

## 1 Introduction

Fungal keratitis (FK) is a severe eye disease that presents substantial risks and challenges, especially for outdoor workers, particularly those working in agricultural areas. Over the past three decades, the number of FK cases has shown a significant rise (Hoffman et al., 2021). Currently, FK accounts for 40%–50% of all cases of microbial keratitis. Alarmingly, it is projected that in less developed nations, more than 600,000 people could suffer from vision loss caused by FK, with outdoor workers being particularly vulnerable (Brown et al., 2022). While being immunocompromised, using contact lenses, experiencing ocular surface trauma (OCD), and suffering vegetative trauma are the primary risk factors for FK, it can also be caused by both filamentous fungi and yeast-like fungi (Ting et al., 2021). Our research team recently isolated a new pathogen, *Coniochaeta hoffmannii*, responsible for causing FK in a 71-year-old patient from a low-income agrarian background (Poomany Arul Soundara Rajan et al., 2023). Individuals who are economically disadvantaged and reside in rural areas with limited access to affordable antifungal medications face the highest risk of developing sight-threatening infections caused by FK (Koffi et al., 2021). The currently used topical therapy antifungals, including amphotericin B, fluconazole, itraconazole, natamycin, and voriconazole, exhibit limited effectiveness in treating FK due to their poor corneal penetration (Sanap et al., 2022). Additionally, the increasing emergence of antifungal antibiotic resistance among FK-causative fungi poses a significant challenge to ophthalmologists (Maharana et al., 2016). Recently several studies have revealed that antibiotics like natamycin and other azoles are becoming ineffective against different fungal pathogens. The exorbitant use of triazole antifungals in agriculture led to the development of azole resistance among several fungal strains. This is a major concern worldwide because even individuals who have never been exposed to these antifungals have been found to carry these resistant strains (Prajna et al., 2022). Considering the complex origin of fungal causatives of this disease, the increasing prevalence of antibiotic-resistant corneal infections, and their projected significant impact on impoverished agricultural communities, the exploration of new therapeutic alternatives and approaches to treating FK is imperative.

In ancient times, folk medicine was relied upon for its effectiveness and affordability in treating illnesses (Krupa et al., 2019). There has been an increased focus on researching bioactive organic therapies, leading to the discovery of over 200,000 medicinal compounds derived from plants. This research is a response to the rising antimicrobial resistance of microbes to conventional drugs (Guimarães et al., 2021). Traditional herbal remedies for eye conditions have been used by ethnic nomadic populations worldwide and extensively studied through ethnobiological research. Throughout history, people have relied on topical medicinal products derived from various plants to treat microbial keratitis. One such plant is *Clitoria ternatea* L., the Asian pigeonwings pea plant. This perennial climber from the Fabaceae family has been widely utilized in ancient medical systems like Ayurveda. It is valued for its numerous medical benefits, including its antidiabetic, analgesic, antipyretic, antioxidant, anticancer, and antimicrobial properties (Singh et al., 2018; Lakshan et al., 2019). *C. ternatea* flower and leaf extracts have effective antimicrobial and antibiofilm properties (Islam et al., 2023). The tribal and nomadic ethnic groups worldwide traditionally use the floral paste of this plant to treat several human ailments, including eye infections, headaches, boils, and skin ailments. Many examples of these traditional uses have been reported, including those by the Irulas of Kodiakkarai, India (Ragupathy and Newmaster, 2009) and several Indonesian communities (Afrianto et al., 2020). The Indian Ayurvedic medicinal system also uses powdered root water mixes for treating eye diseases, headaches, and dyspepsia, which are very common. Their ocular irritation-reducing capability is also widely recognized (Jamil et al., 2018). Their therapeutic effects are believed to be bestowed by the various secondary metabolites like alkaloids, flavonoids, glycosides, resin, saponins, steroids, tannins, and phenols they possess (Tripathi et al., 2023). Hence, the present study aims at assessing the FK therapeutic potential of *C. ternatea* flower extracts through *in vitro* and *in silico* experiments. The current research objective is to determine whether these extracts could be developed and incorporated into safe and effective FK-treating topical agents. Specifically, the study will evaluate the extracts’ ability to inhibit fungal growth, prevent biofilm formation, minimize irritation, penetrate the cornea, and assess their toxicity.

## 2 Materials and methodology

### 2.1 Collection and evaluation of experimental fungal strain’s cultural and biofilm-forming ability

**Figure 1:**
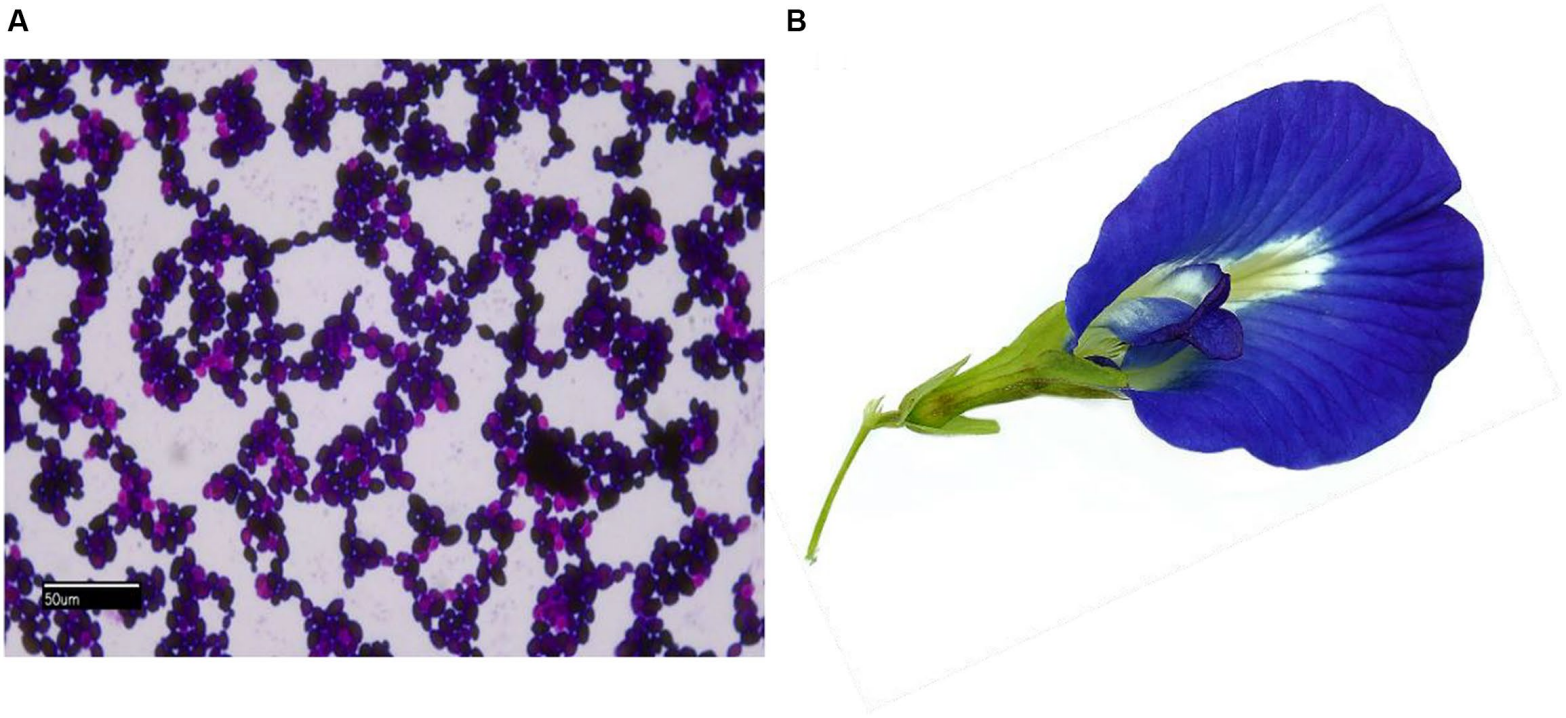
**(A)** Light microscopy gram-stained image (100×) of *C. hoffmannii*; **(B)** photo of *C. ternatea* blue sangupushpam flower.

The study used the biofilm-forming clinical keratitis isolate *C. hoffmannii* (GenBankaccession number: MN453262.1), previously identified (Figure 1A) and isolated from a 70-year-old patient with an agrarian background by our team. The organism was used to evaluate the MIC, MFC, and biofilm inhibitory effects of floral extracts from *C. ternatea*. The growth colony morphology of this strain was examined on Congo red agar. To confirm its biofilm-forming nature, the isolate was streaked on Congo red agar supplemented with Congo red stain, sucrose, and BHI agar (HiMedia Laboratories, India). Afterwards, the inoculated plates were incubated at 27°C for 24–48 h by the method outlined (Khadija et al., 2019). The growth characteristics of the formed colony were observed and documented.

### 2.2 Flower collection and phytocompounds extraction

The blue-colored flowers of the Asian pigeon wings (Figure 1B), locally known as Sangu Pushpam, were collected in and around Tirunelveli, Tamil Nadu, India, at GPS coordinates 8° 44′ 28.3992” N; 77° 41′ 40.6536″ E, between September and December 2022. The plant voucher specimen has been identified as *C. ternatea* L. using the website http://www.worldfloraonline.org. The initial identification was confirmed by the Southern Regional Office of the Botanical Survey of India in Coimbatore. The flowers were collected, dried in the shade, and ground into a coarse powder. The Soxhlet extraction method was used to extract the powdered flower using a sequential gradient of solvents that ranged from nonpolar to polar, including petroleum ether, ethyl acetate, acetone (SRL, Mumbai, India), and water in the ratio 1:10. The solvents were filtered through Whatman™ filter paper and condensed with a help of using a rotary evaporator (RV10 IKA^®^). The resulting solvent-free extracts were stored at 4°C for later use (Rajput et al., 2021).

### 2.3 Antifungal activity

Floral extracts from *C. ternatea* were evaluated for their antifungal activity on potato dextrose agar (PDA) plates following the well diffusion method. *C. hoffmannii* inoculum was cultured in a PDB medium for 48 h at 100 rpm and 27°C. The yeast cell suspension density was adjusted to 0.5 McFarland units using a spectrophotometer (Jasco V-770, Japan). The PDA plates were evenly coated with the prepared inoculum. The solvent-free plant extracts were mixed with DMSO (SD Fine Chemicals Ltd., Mumbai, India) at concentrations (µg/mL) of 1,000, 1,500, 2000, and 2,500. Agar plates were prepared with wells for adding the extracts. Fluconazole (HiMedia Laboratories, Mumbai, India) served as a positive control, whereas the negative control was DMSO. The inhibitory zone’s diameter was measured in millimetres after 48 h of incubation at 27°C (Parveen et al., 2018).

### 2.4 Antifungal MIC and MFC analysis

The MIC and MFC values of *C. ternatea* flower extracts were evaluated using broth dilution and plate methods against *C. hoffmannii*. Four extracts were obtained by diluting concentrations from 0.5 to 2.5 mg/mL in DMSO using two-fold serial dilutions. A culture mixture of 48-h-old *C. hoffmannii* culture at 1.5 × 10^8^ CFU/mL mixed with 100 mL of PDB was prepared. The MIC was determined by adding different amounts of plant extracts (0.25–2.5 mg/mL) to various test tubes. The MIC is the minimum concentration of the extract required to inhibit the growth of the inoculum after a 48-h incubation period at 27°C. To examine MFC, 10 μL of culture from tubes showing no visible growth was added onto a PDA plate. The MFC was calculated as the lowest concentration of extract which allows no fungal growth during incubation on PDA agar plates (Sahal et al., 2020). The floral extract of *C. ternatea* with the highest inhibition rate at the lowest concentration was chosen for further investigation in subsequent tests.

#### 2.4.1 Antibiofilm assay microtiter plate method

A 96-well microtiter plate experiment was employed for the determination CFEA sample’s ability to suppress *C. hoffmannii* biofilm formation. *C. hoffmannii* culture (0.5 McFarland) was diluted with freshly sterilized PDB (1:100). The cell solution (180 μL) was mixed with plant samples (20 μL) of varying MIC concentrations (1/8, 1/4, 1/2, and 1) and dispensed into each well of 96-well plates. The controls are PDB medium with only culture and DMSO without plant extract. The plates were cleaned and stained for 15 min with 0.1% crystal violet after 48 h of incubation at 27°C. After that, they were washed again, and 95% ethanol was used to get rid of well’s leftover stains. Then, the culture was transferred to a new sterile plate, and the OD at 595 nm was measured with a UV–visible spectrometer (Ganesh Kumar et al., 2023). The biofilm inhibition percentage was calculated using the formula [(Control OD570 nm − Treated OD570 nm)/ Control OD570 nm] × 100.

#### 2.4.2 Antibiofilm assay microscopic study

The CFEA’s biofilm inhibitory activity was assessed using the coverslip method. A concentration of 1.5 × 10^8^ CFU/mL of *C. hoffmanii* was prepared in 12-well microtiter plates using sterile PDP (1:100) as a diluent. Different amounts of CFEA (ranging from 1/8 MIC to 1/4 MIC, 1/2 MIC, and 1 MIC) were added to the wells alongside positive and negative controls. Sterile coverslips were placed vertically in the wells, and the plates were incubated for 48 h at 27°C. After incubation, the coverslip was removed, and any unattached planktonic cells were removed by rinsing with sterile PBS. The attached biofilm growth was then stained with 0.1% crystal violet. After removing the excess dye with sterile PBS and air drying the coverslip, the biofilm formation was observed using a light microscope under 100× magnifications, and the outcomes were recorded (Al-Ghanayem, 2022).

### 2.5 Antioxidant test

The DPPH method was utilized to evaluate the CFEA sample’s antioxidant capacity using ascorbic acid as the standard. Varied concentrations of the extract (200, 100, 50, 30, and 10 mg/mL) were mixed with DMSO and an equal volume of DPPH in ethanol (0.004%), and the mixture was kept in the dark for another 30 min. The extract’s free radical scavenging capabilities were assessed by monitoring the purple DPPH color change to yellow and measuring its OD at 517 nm. The IC_50_ value, which represents the amount of extract needed to eliminate 50% of the DPPH, was then calculated using the described method (Rajput et al., 2021).

### 2.6 GCMS phytochemical profiling

The plant CFEA sample’s bioactive compound profiling was carried out using a Shimadzu QP2020 NX GC–MS. The instrument had a single quadrupole, a 190 L/s / 170 L/s (He) differential exhaust turbomolecular pump, a 1,035 kPa pressure range flow controller, and a flame ionization sensor. A 1 mg/mL plant sample was prepared and introduced into the instrument at a split ratio of 1:20 after a 3-min preheating phase at 50°C. The gas chromatograph separation was performed using an Rxi-5sil MS column. The column’s initial temperature was set at 280°C for 2 min, then gradually rose to 330°C at a rate of 12°C every 40 min. The carrier helium gas flow rate was set at 1 mL/min through the injection port maintained at 280°C. For mass spectrometry analysis, an ionization potential of 70 eV was used, along with electron ionization (EI) and transmission line temperatures of 260°C and 280°C, respectively. The sample was fully scanned with a cut-off time of 3 min, from 25 to 500 amu. The gas chromatograph ran for a total of 39 min, with the mass spectrometry analysis taking place between 5 and 40 min. Unidentified compounds within the extract were identified using the Shimadzu GC–MS solution™ Ver.4 software, which utilized the “NIST20R-library” (Syeda and Riazunnisa, 2020).

### 2.7 Functional group and structural detail FTIR spectroscopic analysis

The major functional groups and structural features of the phytoactive chemicals in the plant sample were determined by FTIR spectral analysis. The infrared (IR) spectral details were acquired employing a Nicolet iS5 FT-IR spectrometer, scanning the mid-IR band 32 times at a resolution of 2 cm^−1^. The plant samples were placed in an IR chamber with iD3 ATR attachments before analysis. The resulting spectral details were collected, examined, and processed using Thermo Fischer Scientific OMNIC software, which compared the output spectrum with a reference spectrum from a library to identify the functional groups.

### 2.8 *In silico* toxicity screening and pharmacokinetic prediction

The mutagenic potential, carcinogenicity, and eye irritation ability of the phytocompounds were predicted using the STopTox web portal (Pokharkar et al., 2022). The Swiss-ADME program and ADMET lab 2.0 were used to predict the ADME pharmacokinetic properties. Additionally, the physicochemical properties that influence corneal permeability, such as molecular weight (MW), molecular volume (MV), hydrogen-bond donor (HBD), hydrogen-bond acceptor (HBA), total hydrogen bonds (HBtot), octanol–water partition coefficient (logP), and distribution coefficient logD7.0, were recorded using ADMET lab 2.0 software (Jha et al., 2022).

#### 2.8.1 *In silico* molecular docking analysis

##### 2.8.1.1 Protein preparation

The following receptor proteins were selected based on their functional activity and association with human inflammation: Cellobiose dehydrogenase (Uniprot ID: A0A2I7VT52), Endo-1,4-β-xylanase (Uniprot ID: A0A2I7VT94), and Glucanase (Uniprot ID: A0A2I7VT76). Additionally, human TNF-α (PDB ID: 2az5) and Interleukin IL-1b (PDB ID: 1ITB) were also chosen. These protein structures were obtained in pdb format from the UniProt^1^ and RCBS^2^ databases. To enhance the accuracy of our analysis, unused ligands, co-factors, and water molecules were eliminated using Molegro Molecular Viewer software, and the structures were subsequently exported in PDB format. The proteins were prepared by eliminating co-factors and water molecules as well as adding of polar hydrogen atoms and Kollman charges. This extensive preparation ensures that the target proteins are properly designed and ready for further computational research.

##### 2.8.1.2 Ligand preparation

The ligand structures of 9,9-dimethoxybicyclo[3.3.1]nonane-2,4-dione-; 2,5-O Methylene-D-mannitol; Glycerol 1,2 diacetate; D-Glucitol, 1,4-anhydro; and 1,2,3-Propanetriol were verified using the PubChem database.^3^ Their respective 3D structures were downloaded in Structure Data File (SDF) format. The ligands in sdf format were then converted to pdbqt format for further investigations using the OpenBabel GUI software.

##### 2.8.1.3 Molecular docking

The AutoDock 1.5.6, an automated protein-ligand docking tool, was used to predict the binding affinity between phytochemical ligands from plant samples and five protein receptors. Fluconazole (PubChem ID: 3365), a commonly available commercial antifungal drug used as a topical azole, was used as a positive control. The grid parameter file (GPF) with a grid box was built using the Lamarckian Genetic algorithm. The constructed grid box’s dimensions (spacing; npts (x, y, z); center (x, y, z)) for the selected receptors are as follows: A0A2I7VT52 (0.897, (18.415, −15.886, −3.835), (126,110,110)); A0A2I7VT94 (0.919, (−6.622,-18.687,-28.162), (90,92,112)); A0A2I7VT76 (0.731, (3.210, 5.88, −14.513), (110, 96, 112)); 2az5 (0.936, (13.680, 71.619, 27.007), (92, 76, 80)); 1ITB (0.936, (39.651, 4.651, 14.919), (126, 80, 48)). MD studies were conducted using the Lamarckian genetic algorithm (LMA) and an empirical free energy function as outlined by Forli et al. (2016). A population size of 300 and 50 iterations of the genetic algorithm were employed to create the docking parameter file (DPF) using AutoDock Tools (ADT). The complexes generated from the lowest-energy conformation in each run were organized into clusters (Sunny et al., 2022).

#### 2.8.2 Molecular mechanics with generalized born and surface area (MMGBSA)

The binding free energy of the receptor, ligand and their complexes were calculated using the MM-GBSA module of the software (Schrödinger suite Schrödinger, New York, 2021). The OPLS4 force field and rotamer algorithm were employed to assess the relative energy of these complexes. The free-binding energy equation is expressed as follows:


$$\Delta G_{\mathrm{bind}}=\Delta G_{\mathrm{complex}}:\left(\Delta G_{\mathrm{protein}}+\Delta G_{\mathrm{ligand}}\right)$$

A lower negative score indicates a stronger binding energy.

#### 2.8.3 Molecular dynamics simulations (MDS)

MDS were performed on the top-ranked receptor complexes, including cellobiose dehydrogenase, endo β 1,4 xylanase, glucanase, TNF-α, and Interleukin IL-1b, using the Schrödinger software’s Maestro platform Desmond tool. The binding scores from the MDS studies were examined for the top-ranked ligands, specifically 2,5-O-Methylene-D-mannitol and 9,9-dimethoxy bicyclo [3.3.1] nonane-2,4-dione. The receptors were preprocessed, hydrogen bond optimized, and OPLS4 force field minimized before MDS. A hydration model was created by solvating the receptor and ligand combination in a 3D orthorhombic box using the SPC water model. The MD simulations were run for 150 ns with 1,000 projections under the NPT ensemble, while maintaining constant temperature (T), pressure (P), and number of atoms (N). The model system was relaxed using standard Desmond settings before the simulation began. A simulation interactions diagram was used to analyze the complex’s stability, revealing RMSD (root mean square distance) data. Throughout the simulation period, protein–ligand connections were determined using hydrogen bonds, hydrophobic interactions, ionic interactions, and water bridges (Schrödinger: Desmond Molecular Dynamics System, NY, 2021) as described (Gunaseelan et al., 2022).

### 2.9 *In vitro* cytotoxicity cell line assay

The CFEA phytochemical’s toxicity on SIRC (NCCS, Pune, India) cells was assessed using MTT colorimetric assay. DMEM media (Gibco, United States) with FBS (10%) and antibiotic solution (1%) supplementation was used growing the cells until reaching 1 × 10^5^ cells/mL concentration in a 96-well culture plate. After incubation at 37°C for 24–48 h, the wells were rinsed with sterile PBS and treated with various amounts of plant samples in a serum-free DMEM medium. The plate was then incubated for 24 h at 37°C with 5% CO_2_. Following that, 10 μL of MTT (5 mg/mL) was added to each well and incubated until a purple precipitate appeared under an inverted microscope. The well plates were washed with 1X PBS after the spent culture and MTT were removed. The formed formazan crystals were dissolved by adding 100 μL of DMSO and shaking the plate for 5 min. Cell viability percentages were calculated by quantifying the MTT reduction to formazan crystal through measuring the OD values at 570 nm using the microplate reader (Thermo Fisher Scientific, United States) as described by Chainumnim et al. (2022).


Cellviability%=Testabsorbance/Controlabsorbance×100.

### 2.10 *In vitro* chorioallantoic egg eye irritation (CAE-EI) assay

The *in vitro* hen’s egg chorioallantoic membrane-embryo irritation (CAE-EI) experiment, as described by Rangel et al. (2020) was utilized to assess the CFEA sample’s potential for causing ocular irritation. Fertilized White Leghorn chicken eggs were obtained from the Veterinary College and Research Institute in Tirunelveli after being incubated for 9 days. The eggshells were sterilized with ethanol, and a rotary dentist saw blade was used to carefully remove them without harming the chorioallantoic membrane (CAM). To test for eye irritation, a solution containing 0.3 mL of the extracted sample in a 0.5% DMSO solution was applied to the CAM. The positive control used was 0.1 N NaOH, and the negative control was 0.9% NaCl. Following the ICCVAM methodology, the CAM reactions such as vascular lysis or coagulation were observed after 5 min. The resulting irritation scores, ranging from 0 to 21, were assigned to categorize irritants as none (0–0.09), weak (1–4.9), medium (5–9.9), and strong (10–21), and recorded for analysis (Luepke and Kemper, 1986).

### 2.11 Statistical analysis

The data from the experiments performed thrice were reported as mean with ± standard deviation. SPSS 22.0 was used to carry out a one-way ANOVA analysis and the *p*-values less than 0.05 were considered as statistically significant.

## 3 Results

### 3.1 Antifungal effect of CFEA on FK clinical isolate

**Figure 2:**
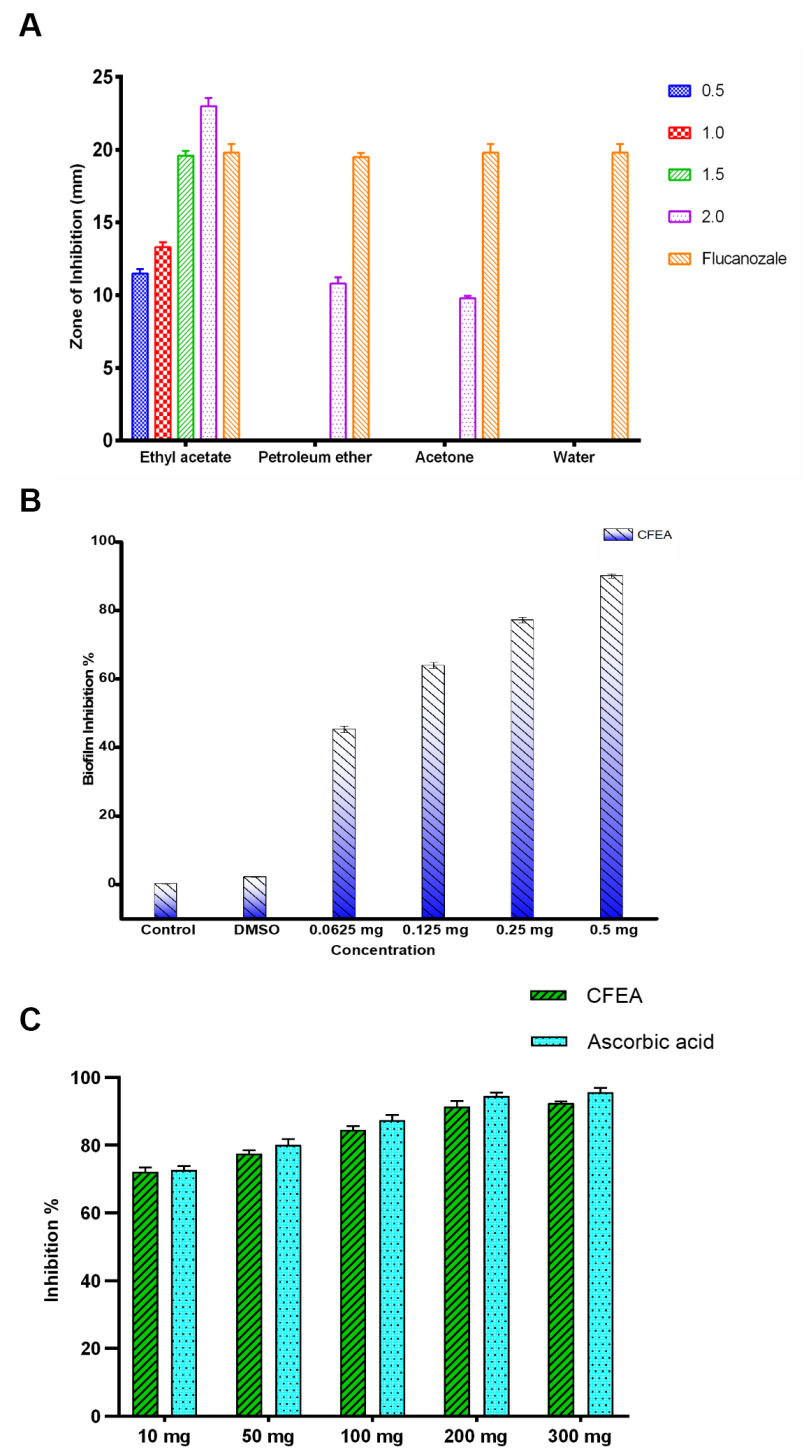
The graph represents **(A)** antifungal activity of *C. ternatea* L. flower extracts against *C. hoffmannii* at varying concentrations. **(B)** Antibiofilm inhibition percentage of CFEA against *C. hoffmannii*. **(C)** CFEA antioxidant activity at various concentrations and its comparison with standard ascorbic acid.

Ongoing research is being conducted on the potential therapeutic benefits of the blue flowers of *C. ternatea*, which have traditionally been used to treat eye ailments. The study found that *C. ternatea* flower extracts had significant antifungal activity against *C. hoffmannii*, a clinical isolate fungus. CFEA showed the strongest inhibitory effect at the lowest concentration. The water extract had no inhibitory effects, while petroleum ether and acetone extracts had reduced inhibitory effects. The average inhibition zone of the active extracts ranged from 9.83 ± 0.28 to 23 ± 1.0 mm. The CFEA extract showed the most potent antifungal activity, ranging from 11.50 ± 0.50 to 23 ± 1.0 mm (Figure 2A). The DMSO used as a negative control had no inhibitory effects. In contrast, the positive control, fluconazole, resulted in an inhibition zone of 23 ± 0.5 mm. The results demonstrate that *C. hoffmanni* is more susceptible to CFEA extract than other extracts, having MIC and MFC values of 0.5 and 1 mg mL^−1^, respectively.

### 3.2 Antibiofilm activity of CFEA

**Figure 3:**
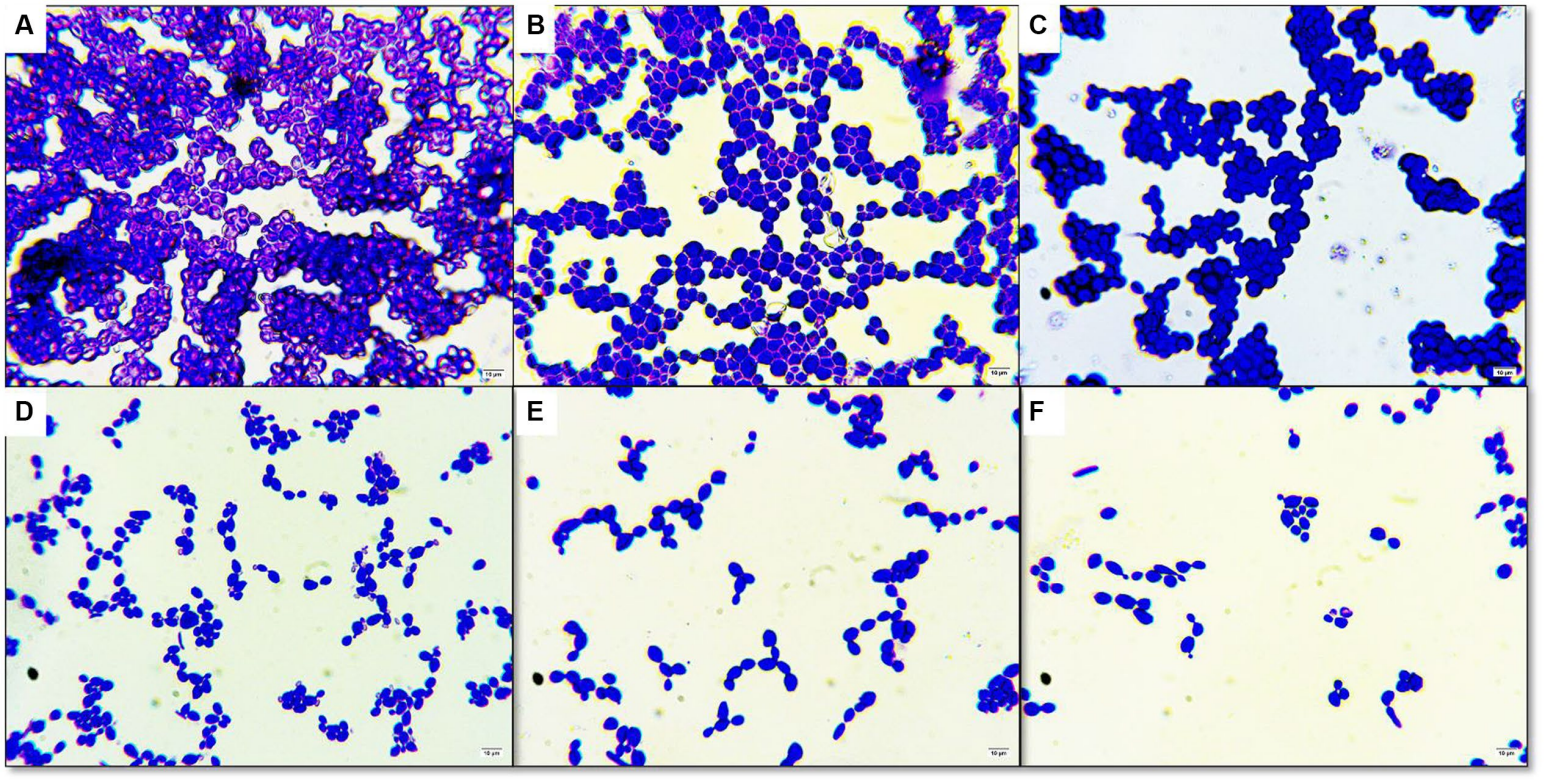
Antibiofilm activity under light microscope in 100× magnification **(A)** control without plant extract **(B)** DMSO only **(C)** treated with 1/8 MIC of CFEA **(D)** treated with 1/4 MIC of CFEA **(E)** treated with 1/2 MIC of CFEA **(F)** treated with 1 MIC of CFEA.

Under an optical microscope, living cells stained with crystal violet were observed as part of the antibiofilm assay (Figure 3). The ability of CFEA to effectively inhibit biofilms was demonstrated by exposing *C. hoffmannii* to varying doses of the compound (Figure 2B). The intensity of biofilm formation decreased as the concentration of CFEA increased, indicating a dose-dependent relationship for inhibiting biofilm formation. The least effective MIC doses for inhibiting biofilm growth were 1/4 and 1/2.

### 3.3 Quantified antioxidant activity of CFEA

At concentrations (mg/mL) of 10, 50, 100, 200, and 300, the antioxidant levels of CFEA were observed to increase by 72%, 78%, 83%, 91%, and 92%, respectively. These results closely matched those of the control group, with ascorbic acid indicating the strong antioxidant capacity of CFEA (Figure 2C). The IC_50_ of CFEA was found to be 1 mg, demonstrating its ability to reduce DPPH radical scavenging activity by 50% at this concentration. This study highlights the impressive antioxidant effects of CFEA and its effectiveness in eliminating DPPH radicals.

### 3.4 CFEA bioactive components GC–MS profiling

**Figure 4:**
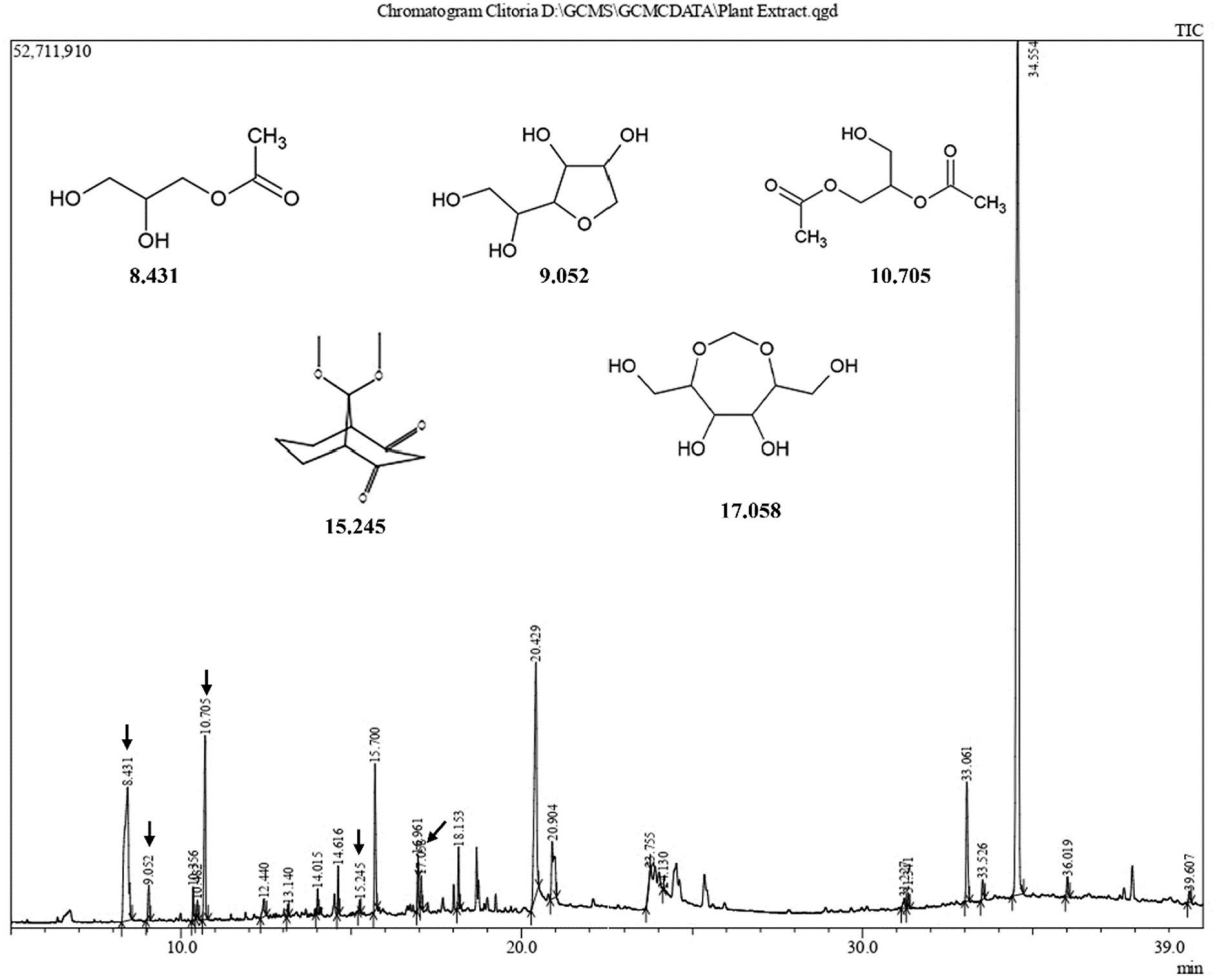
GC–MS chromatogram of CFEA.

Upon comparing the CFEA extract to the “NIST20R library,” the GCMS spectral data analysis revealed the presence of 23 phytochemicals in different quantities. The GC–MS chromatogram (Figure 4) indicates 23 peaks in the CFEA. These include compounds such as Hexanoic acid, 2-ethyl-, anhydride (49.18%); n-Hexadecanoic acid (11.86%); 1,2,3-Propanetriol, 1-acetate (11.71%); Glycerol 1,2-diacetate (5.58%); Hexadecanoic acid, octyl ester (4.03%); Phthalic acid, diethyl ester (3.89%); 1-Hexacosene (3.70%); 9-Tricosene, (Z)- (1.26%); D-Glucitol, 1,4-anhydro- (1.04%); Phenol, 2,4-bis (1,1-dimethyl ethyl)- (0.99%); Sorbitol (0.87%); Benzofuran, 2,3-dihydro- (0.77%); 2,5-O-Methylene-D-mannitol (0.73%); Isopropyl hexacosyl ether (0.66%); Phthalic acid, bis(2-ethylhexyl) ester (0.65%); Hexadecanoic acid, 3-hydroxy-, methyl ester (0.57%); 5-Hydroxymethyl-2-furaldehyde (0.47%); Propanoic acid, 2-methyl-, nonyl ester (0.47%); 2-Methylhexacosane (0.45%); 1-Docosanol, methyl ether (0.44); Dotriacontyl isopropyl ether (0.43%); 9,9-dimethoxy bicyclo [3.3.1] nonane-2,4-dione- (0.27%) and Sulfurous acid, dodecyl 2-propyl ester (0.23%).

### 3.5 Chemical bonding functional groups of CFEA

**Figure 5:**
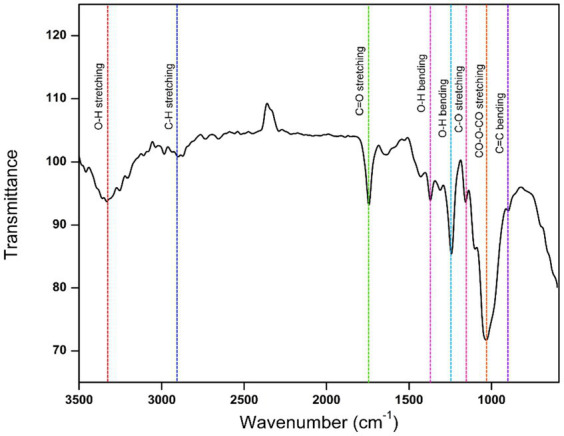
FTIR spectrum showing functional groups of CFEA.

The CFEA FTIR spectrum revealed several functional groups of CFEA phytocompounds. Major absorption peaks were found at specific wave numbers (cm^−1^): 851, 1,047, 1,238, 1,371, 1,438, 1739, 2,987, and 3,328, which confirms the presence of significant chemical bonding groups. The peak at 1,047 cm^−1^ corresponds to the CO-O-CO bond stretch in the anhydride group, while the peak at 851 cm^−1^ represents the C=C alkene group bend. Peaks at 1,371 cm^−1^, 1,438 cm^−1^, and 3,328 cm^−1^ indicate the O-H groups of phenols, carboxylic acids, and alcohols, respectively. Additionally, peak values at 1,238 cm^−1^, 1,739 cm^−1^, and 2,987 cm^−1^ represent the C-O, C=O, and C-H stretches of alkyl aryl ethers, carbonyl esters, and alkane groups (Figure 5).

### 3.6 *In silico* predicted CFEA corneal permeability and druggable pharmacological properties

The corneal permeability and other pharmacological parameters of compounds are crucial for formulating topical ophthalmic medications. This data includes MW, MV, HBD, HBA, total hydrogen bonds (HBtot), Log P, and Log D. Some CFEA phytocompounds adhere to the Lipinski rule of drug-likeness, showing potential for conversion into orally bioavailable medications and topical treatments for FK. However, some CFEA compounds violate Lipinski’s rule, and some are predicted to have ocular irritation, carcinogenic, mutagenic, or mutagenic potential. However, some compounds of CFEA violate Lipinski’s rule. They include Sorbitol, 9-tricosene, (Z)-, 1-hexacosene, 2-methylhexacosane, Hexadecanoic acid, octyl ester, bis(2-ethylhexyl) phthalate, and Isopropyl hexacosyl ether, each with one violation and Dotriacontyl isopropyl ether with two violations. The compounds Glycerol 1,2-diacetate, D-Glucitol, 1,4-anhydro-, 1,2,3-Propanetriol, 1-acetate, 9,9-dimethoxybicyclo[3.3.1]nonane-2,4-dione-, and 2,5-O Methylene-D-mannitol are designated as corneal-permeable, druggable phytocompounds based on their properties. Table 1 provides each compound’s physiochemical and pharmacokinetic properties.

**Table 1:** GC–MS analysis of phytocompounds present in CFEA, their physiochemical, and pharmacokinetic properties.

Sl. no	Name of the compound	PubChem ID	RT	MF	MW	Area %	HBA	HBD	HB_tot_	Log P	Log D (pH 7.4)	Mut Pot	Car	EI	Lipinski rule-based probability
1	Hexanoic acid, 2-ethyl-, anhydride	161,921	34.554	C_16_H_30_O_3_	270.41	49.18	3	0	3	5.297	4.792	__	__	__	Druggable
2	n-Hexadecanoic acid	985	20.429	C_16_H_32_O_2_	256.24	11.86	2	1	3	6.732	3.235	+	__	__	Druggable
3	1,2,3-Propanetriol, 1-acetate	33,510	10.705	C_5_H_10_O_4_	134.13	11.71	5	1	6	−0.896	−1.143	__	__	__	Druggable
4	Glycerol 1,2-diacetate	66,021	8.431	C_7_H_12_O_5_	176.17	5.58	4	2	6	−0.595	−0.262	__	__	__	Druggable
5	Hexadecanoic acid, octyl ester	85,651	33.061	C_24_H_48_O_2_	368.6	4.03	2	0	2	9.925	4.741	__	__	__	Non-druggable
6	Phthalic acid, diethyl ester	6,781	15.700	C_12_H_14_O_4_	222.24	3.89	3	1	4	2.681	2.736	+	+	+	Druggable
7	1-Hexacosene	29,303	20.904	C_26_H_52_	364.7	3.70	0	0	0	12.026	4.936	__	__	__	Non-druggable
8	9-Tricosene, (Z)-	5,365,075	18.153	C_23_H_46_	322.6	1.26	0	0	0	10.701	5.146	__	__	__	Non-druggable
9	D-Glucitol, 1,4-anhydro-	10,953,859	9.052	C_6_H_12_O_5_	164.16	1.04	5	4	9	−2.089	−1.696	__	__	__	Druggable
10	Phenol, 2,4-bis (1,1-dimethyl ethyl)-	7,311	14.616	C_14_H_22_O	206.32	0.99	1	1	2	4.832	4.333	__	__	+	Druggable
11	Sorbitol	5,780	12.440	C_6_H_14_O_6_	182.17	0.87	6	6	12	−2.608	−2.328	__	__	__	Non-druggable
12	Benzofuran, 2,3-dihydro-	10,329	10.356	C_8_H_8_O	120.15	0.77	1	0	1	2.318	2.236	__	__	+	Druggable
13	2,5-O-Methylene-D-mannitol	99,468	17.058	C_7_H_14_O_6_	194.18	0.73	6	4	10	−2.050	−1.874	__	__	__	Druggable
14	Isopropyl hexacosyl ether	243,696	36.019	C_29_H_60_O	424.7861	0.66	1	0	1	12.272	4.609	__	__	__	Non-druggable
15	Phthalic acid, bis(2-ethylhexyl) ester	8,343	33.526	C_24_H_38_O_4_	390.6	0.65	4	0	4	7.337	5.716	+	+	__	Non-druggable
16	Hexadecanoic acid, 3-hydroxy-, methyl ester	103,553	14.015	C_17_H_34_O_3_	286.4	0.57	3	1	4	5.648	3.827	__	__	__	Druggable
17	5-Hydroxymethyl-2-furaldehyde	237,332	10.482	C_6_H_6_O_3_	126.11	0.47	3	1	4	0.148	0.544	+	+	+	Druggable
18	Propanoic acid, 2-methyl-, nonyl ester	8,139	16.961	C_13_H_26_O_2_	214.34	0.47	2	0	2	5. 231	3.975	__	__	__	Druggable
19	2-Methylhexacosane	150,931	31.341	C_27_H_56_	380.7	0.45	0	0	0	12.557	5.432	__	__	__	Non-druggable
20	1-Docosanol, methyl ether	87,077,550	31.227	C_23_H_48_O	340.6	0.44	1	0	1	10.138	4.653	__	__	__	Druggable
21	Dotriacontyl isopropyl ether	91,692,940	39.607	C_35_H_72_O	508.9	0.43	1	0	1	14.523	5.373	__	__	__	Non-druggable
22	9,9-dimethoxy bicyclo [3.3.1] nonane-2,4-dione-	537,288	15.245	C_11_H_16_O_4_	212.24	0.27	4	0	4	0.971	−0.191	__	__	__	Druggable
23	Sulfurous acid, dodecyl 2-propyl ester	6,420,354	13.140	C_15_H_32_O_3_S	292.5	0.23	3	0	3	5.849	4.470	__	__	__	Druggable

RT, Retention time; MF, molecular formula; MW, molecular weight; HBA, Hydrogen bond acceptor; HBD, Hydrogen bond donor; HB_tot_, Total Hydrogen bond; Log P, partition coefficient; Log D, distribution coefficient; MP, Mutagenic potential; Car, Carcinogenicity; EI, Eye irritation.

#### 3.6.1 CFEA phytocompounds interaction with fungal virulence and human inflammatory proteins

**Figure 6:**
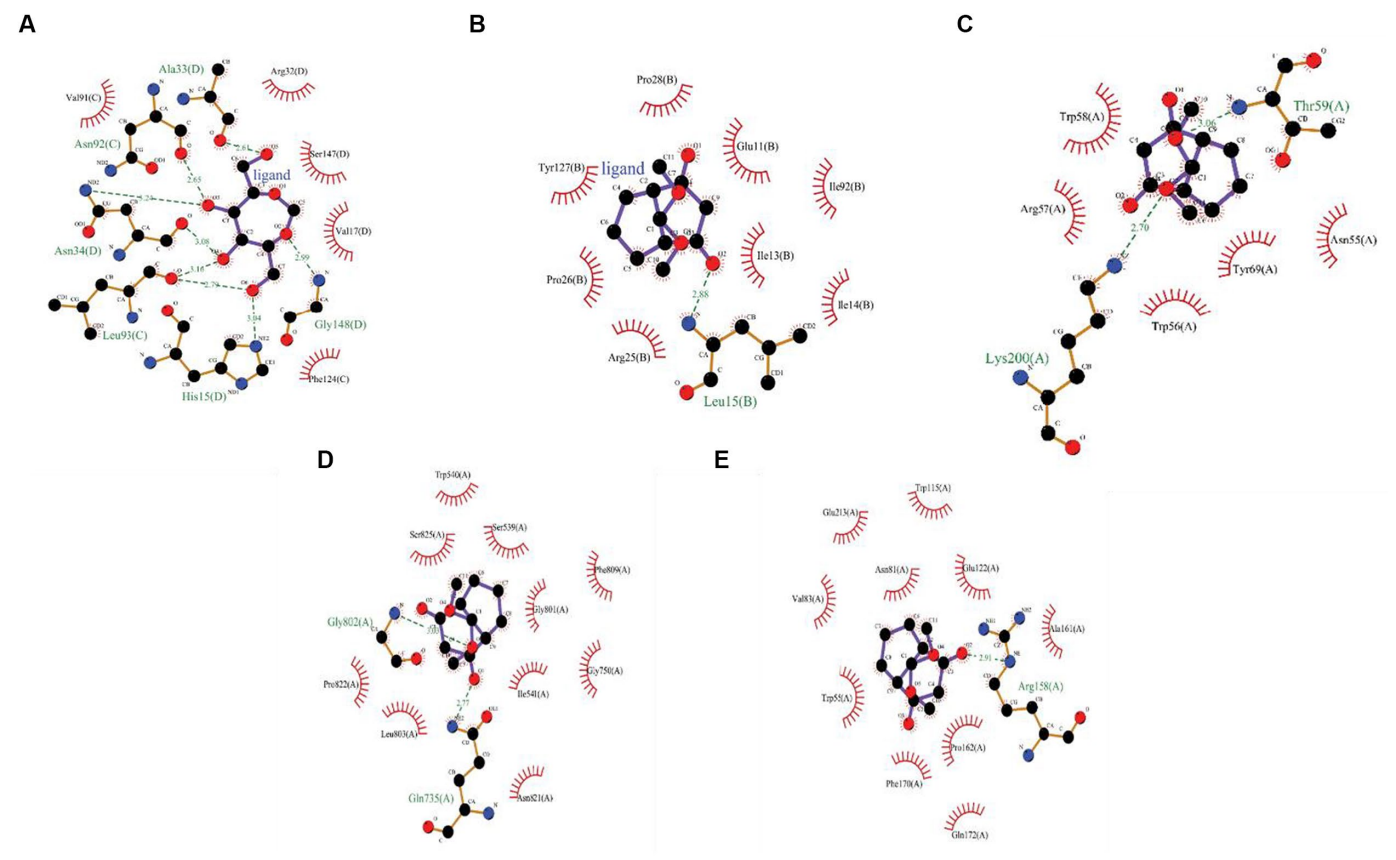
**(A)** 2D interactions between 2,5-O-Methylene-D-mannitol with human TNF-α, **(B)** 2D interactions between 9,9-dimethoxybicyclo[3.3.1]nonane-2,4-dione-, with human Interleukin IL-1b,2D interactions between 9,9-dimethoxybicyclo[3.3.1]nonane-2,4-dione-, **(C)** with Cellobiose dehydrogenase, **(D)** Endo β 1,4 xylanase, and **(E)** Glucanase.

The CFEA druggable phytocompounds, such as 9,9-dimethoxy bicyclo [3.3.1] nonane-2,4-dione; 2,5-O Methylene-D-mannitol; Glycerol 1,2-diacetate; D-Glucitol, 1,4-anhydro- and 1,2,3-Propanetriol, 1-acetate, showed significant affinity when docked with fungal enzymes Cellobiose dehydrogenase, Endo-1,4-β-xylanase, and Glucanase, with binding score ranging from −2.05 kcal/mol to −5.19 kcal/mol. Among these compounds, 9,9-dimethoxy bicyclo [3.3.1] nonane-2,4-dione- exhibited the highest binding values of −5.19 kcal/mol, −5.06 kcal/mol, and −4.7 kcal/mol with Cellobiose dehydrogenase, Endo β 1,4 xylanase, and glucanase, respectively (Figure 6). The amino acids involved in the hydrogen bond interaction of 9,9-dimethoxy bicyclo [3.3.1] nonane-2,4-dione- with the three amino acids were Gln735, Gly802, Arg158, Thr59, and Lys200. The inhibition constants for these interactions were 156.22 μM, 195.21 μM, and 356 μM, respectively. These druggable compounds also showed good *in silico* interaction with corneal inflammation-inducing proteins human TNF-α and Interleukin IL-1b, with binding energies from −5.41 kcal/mol to −8.56 kcal/mol. Among these compounds, 2,5-O-Methylene-D-mannitol and Bicyclo [3.3.1] nonane-2,4-dione, 9,9-dimethoxy- exhibited the lowest binding values with the targeted proteins. Table 2 shows the binding score of five ligands with fungal virulence and inflammatory receptorsprotein, as well as the distances between Van der Waal’s interaction bonds and the number of hydrogen bonds formed. These lowest binding scores show that the CFEA compounds are more efficient in interacting and suppressing the fungal virulence and inflammatory proteins, thereby reducing the fungal pathogenicity and corneal inflammation. The calculated binding free energy of the docked complexes as calculated by MM-GBSA suggests a strong ligand-receptor affinity. Specifically, the relative binding free energies of glucanase and cellobiose dehydrogenase with 9,9-dimethoxy bicyclo[3.3.1]nonane-2,4-dione are −8.41765 kcal/mol and − 38.2048 kcal/mol, respectively. Additionally, the binding free energy between Endo β 1,4 xylanase and 2,5-O-Methylene-D-mannitol is determined to be −39.7775 kcal/mol.

**Table 2:** Results of molecular docking analysis showing docking scores, inhibition constant, and interacting amino acid residues.

Sl. no	Protein name	Ligand	Binding energy (kcal/mol)	Inhibition constant	No hydrogen bonds	H bond-forming residues	Distance H-A (A°)	Hydrophobic residues
1	Cellobiose dehydrogenase	Glycerol 1,2-diacetate	2.25	22.37 mM	02	Gln735, Gly802, Ser825	2.98, 3.03, 2.91	Ile541, Pro822, Cys817, Leu803, Gly801, Val819, Gly469, Phe809, Gly750,
2		D-Glucitol, 1,4-anhydro-	−2.38	18.0 mM	03	Thr114, Leu134, Val229	3.14, 2.81, 3.06	Thr133, Thr228
3		1,2,3-Propanetriol, 1-acetate	−1.81	46.8 mM	01	Leu134	3.02	Thr144, Thr228, Val229, Gln136
4		9,9-Dimethoxybicyclo (3.3.1) nona-2,4-dione-	−5.19	156.22 μM	01	Gln735, Gly802	2.77, 3.03	Trp540, Ser825, Ser529, Gly801, Phe809, Gly750, Ile541, Pro822, Leu803, Asn821
5		2,5-O-Methylene-D-mannitol	−2.8	8.83 mM	01	Met124, His199, Try122	3.02, 3.19, 2.85	Trp88, Met97, Gln198, Leu196, Ala197, Asp123,
6		Fluconazole	−7.27	4.69 μM	02	Gly339, Thr504	3.15, 3.00	Ser501, Ala502, Gly260, Gly503, Gly338, Trp723, Phe505, Pro770, Asn721, Ala759
7	Endo β 1,4 xylanase	Glycerol 1,2-diacetate	−2.05	31.17 mM	01	Try52, Thr35, Gln37	2.84, 3.29, 3.11	Glu34, Arg36, Asn47, Asn66, Ile39, Try46
8		D-Glucitol, 1,4-anhydro-	−2.33	19.64 mM	01	Glu34, Asn48	2.82, 3.09	Arg36
9		1,2,3-Propanetriol, 1-acetate	−2.4	17.28 mM	03	Asn89, Pro90, Arg94	2.81, 2.86, 3.07	Tyr46, Trp88
10		9,9-Dimethoxybicyclo (3.3.1) nona-2,4-dione-	−5.06	195.21 uM	01	Arg158	2.91	Trp115, Glu213, Val83, Trp55, Asn81, Glu122, Ala161, Pro162, Phe170, Gln172
11		2,5-O-Methylene-D-mannitol	−3.21	4.46 mM	03	Asn47, Glu34, Asp32	2.92, 2.94, 2.70	Leu33, Leu67, Asn48, Asn66
12		Fluconazole	−6.63	13.87 uM	01	Gln172, Glu122, Tyr124	2.94, 2.71, 3.26	Trp174, Tyr113, Glu213, Val83, Trp55, Asn81, Pro162, Arg158
13	Glucanase	Glycerol 1,2-diacetate	−3.05	5.79 mM	02	Lys200, Thr59, Gly71, Asn219	2.87, 2.87, 3.11, 3.08	Trp58, Tyr69, Arg57, Trp56, Asn55
14		D-Glucitol, 1,4-anhydro-	−2.68	1.078 mM	03	Arg114, Asp116, Asp435, Lys112	2.76, 3.16, 3.03, 2.63	Lys434, Val115,
15		1,2,3-Propanetriol, 1-acetate	−2.49	14.95 mM	03	Gly93, Trp58, Try100	2.86, 3.23, 2.74	Trp56, Thr99, Arg57,
16		9,9-Dimethoxybicyclo (3.3.1) nona-2,4-dione-	−4.7	356.19 uM	02	Thr59, Lys200	3.06, 2.70	Trp58, Arg57, Asn55, Tyr69, Trp56
17		2,5-O-Methylene-D-mannitol	−3.94	1.29 mM	05	Gly210, Val208, Trp211, Asp73	3.05, 2.87, 2.82, 3.07	Trp74, Lys76, Cys79, Glu209
18		Fluconazole	−8.25	901.17 nM	03	His117, Trp56, Thr59, Lys200	2.79, 3.08, 2.95, 2.84	Thr121, Tyr100, Asn122, Asn219, Asn55, Tyr69, Arg57, Trp58, Thr99
19	TNF-α	Glycerol 1,2-diacetate	−6.65	13.44 μM	03	Ala33, Val150, Gln149	3.07, 2.97, 2.87	Val91, Ser147, Arg32, Gly148, Phe124, Asn34, Leu93, Asn92, Ala18, Val17
20		D-Glucitol, 1,4-anhydro-	−6.80	10.28 μM	04	Ser147, Gly148, Glu146, Ala18	2.55, 3.11, 3.00, 2.93	Arg32, Pro20, Gln149, Phe144, Val150
21		1,2,3-Propanetriol, 1-acetate	−6.55	15.79 μM	03	Gly148, Ala18, Gln149, Val150	2.97, 2.94, 3.17, 2.85	Pro20, Ser147, Arg32, Val17, Phe144, Glu146.
22		9,9-Dimethoxybicyclo (3.3.1) nona-2,4-dione-	−6.37	21.56 μM	03	Arg82, Asn34, Gln125	3.34, 2.73, 3.19	Ala33, Ala35, Arg32, Leu36
23		2,5-O-Methylene-D-mannitol	−8.56	533.88 nM	06	Ala33, Asn92, Asn34, Leu93, His15, Gly148	2.61, 2.65, 3.24, 3.16, 3.04, 2.99	Val91, Arg32, Ser147, Val17, Phe124
24		Fluconazole	−7.92	1.55 μM	01	Tyr151, Leu120, Tyr151	3.10, 2.59, 2.89	Leu57, Tyr119, Tyr59, Gly121, Ser60, Tyr119, Ser60
25	Interleukin IL-1b	Glycerol 1,2-diacetate	−6.24	26.64 μM	03	Met128, Gln14, Cys125	3.21, 2.75, 2.67	Ala127, Glu129, Gln126, Glu128, Pro126, Asp162, Lys16, Tyr127, Val124, His30
26		D-Glucitol, 1,4-anhydro-	−6.19	29.98 μM	03	Leu80, Leu26, Val132	2.67, 3.25, 2.92	Pro131, Leu82, Glu25, Gln81
27		1,2,3-Propanetriol, 1-acetate	−5.41	169.88 μM	02	Trp134, Lys161	3.04, 2.87	Leu141, Val160, Gly159
28		9,9-Dimethoxybicyclo (3.3.1) nona-2,4-dione-	−8.11	1.14 μM	01	Leu15	2.88	Try127, Pro28, Pro26, Arg25, Glu1, Ile13, Ile92, Ile14
29		2,5-O-Methylene-D-mannitol	−7.23	5.05 μM	04	Pro26, Ile13, Leu15, Arg25	2.79, 2.84, 3.01, 2.96	Glu11, Pro28, Try127, Pro26, Ile13, Ser93, Ile14, Ile92
30		Fluconazole	−7.64	2.5 μM	01	Tyr127	2.95	Glu129, Ile13, Phe130, Pro26, Glu128, Pro28, Ile92, Arg25, Asn129, Leu29, Leu15

#### 3.6.2 Conformation of stability of docking complexes by molecular dynamics simulations

**Figure 7:**
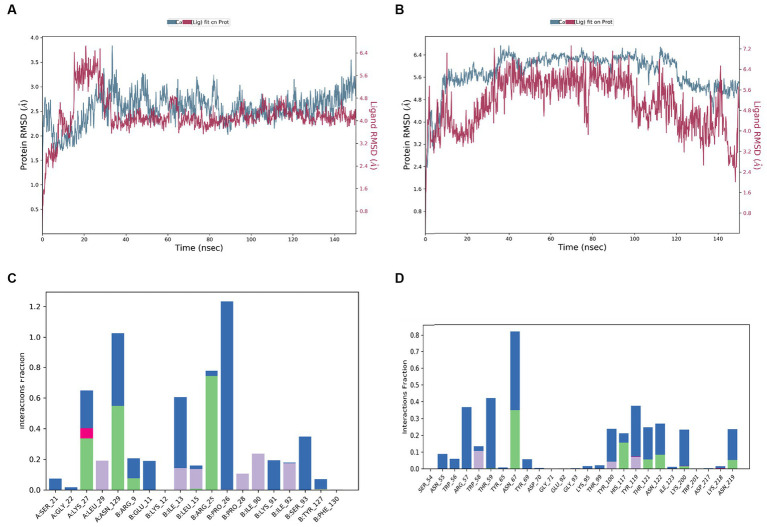
RMSD values during the simulation of complex of **(A)** 9,9-dimethoxybicyclo[3.3.1]nonane-2,4-dione-, with human Interleukin IL-1b, **(B)** 9,9-dimethoxybicyclo[3.3.1]nonane-2,4-dione-, with glucanase; protein ligand interaction histogram of **(C)** 9,9-dimethoxybicyclo[3.3.1]nonane-2,4-dione-, with human Interleukin IL-1b, **(D)** 9,9-dimethoxybicyclo[3.3.1]nonane-2,4-dione-,with glucanase.

A 150 ns MDS was performed on the top docked complexes to evaluate the stability of the simulated systems and analyze the hydrogen bonds, hydrophobic, ionic, and water bridge interactions. The results indicate strong structural stability and interactions within the docking complexes, as evidenced by the calculated RMSD, RMSF, and protein-ligand contact. Figures 7A,B illustrate the RMSD of Interleukin IL-1b with the 2,5-O-Methylene-D-mannitol complex and glucanase with the 9,9-Dimethoxybicyclo (3.3.1) nona-2,4-dione-complex. The RMSD values for the ligand and receptor fluctuated around 3 Å throughout the simulation, indicating the degree of stability of the complex. Strong and sustained contact between the ligand and protein is evident. According to Figure 7C, the residues Lys27, Leu29, Asn129, Arg9, Glu11, Ile13, Leu15, Arg25, Pro26, Ile90, Lys91, and Ser93 exhibit hydrogen bond, hydrophobic, and water bridge interactions. Additionally, Asn55, Trp56, Arg57, Trp58, Thr59, Asn67, Tyr100, His117, Tyr119, Thr121, Asn122, Lys200, and Asn219 are shown in Figure 7D to interact through hydrogen bond, hydrophobic, and water bridges, demonstrating the stability of the complex.

#### 3.6.3 CFEA eye-irritating and cytotoxicity potency

**Figure 8:**
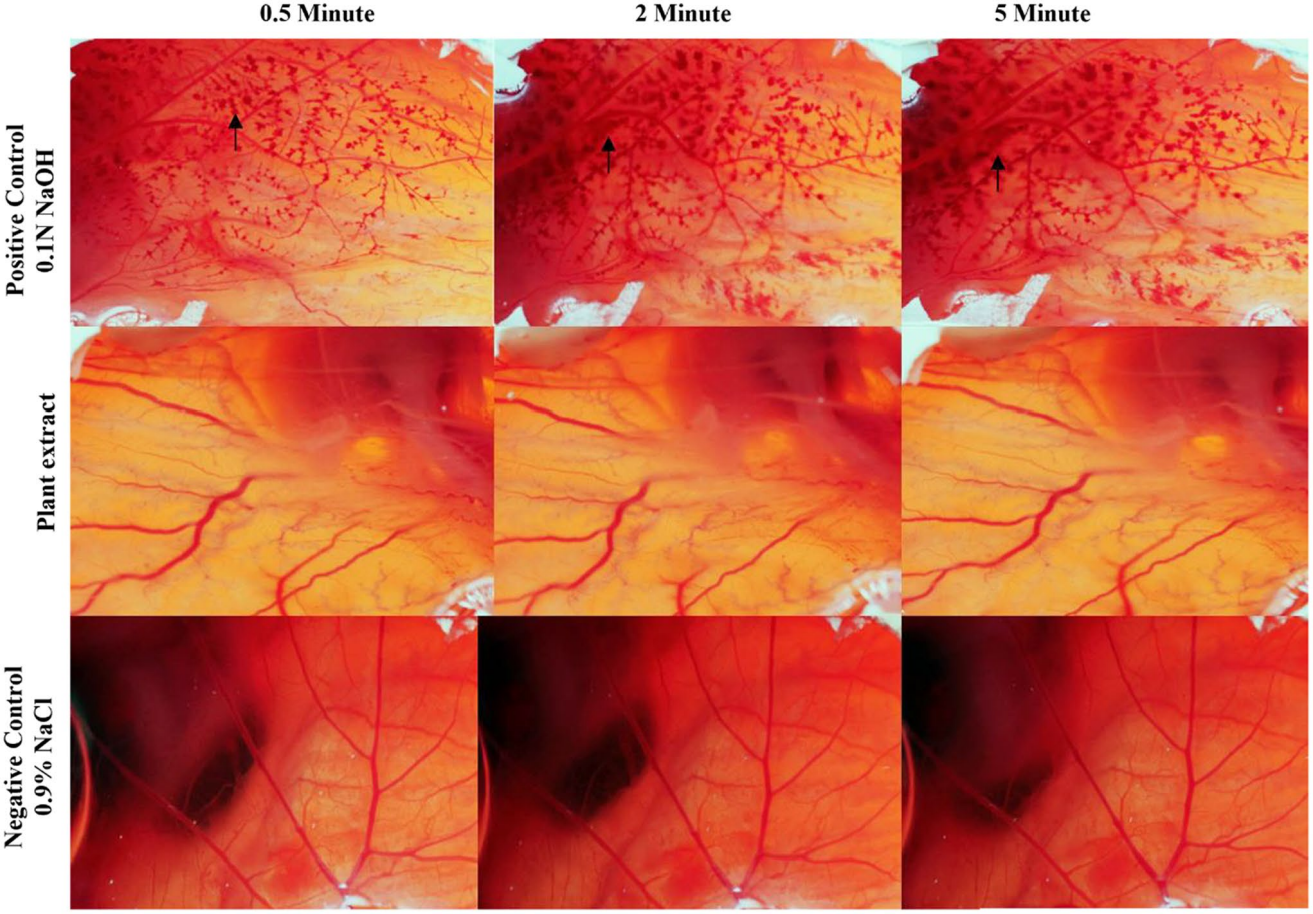
CAE-EI assay to test the ophthalmic irritation of CFEA.

**Figure 9:**
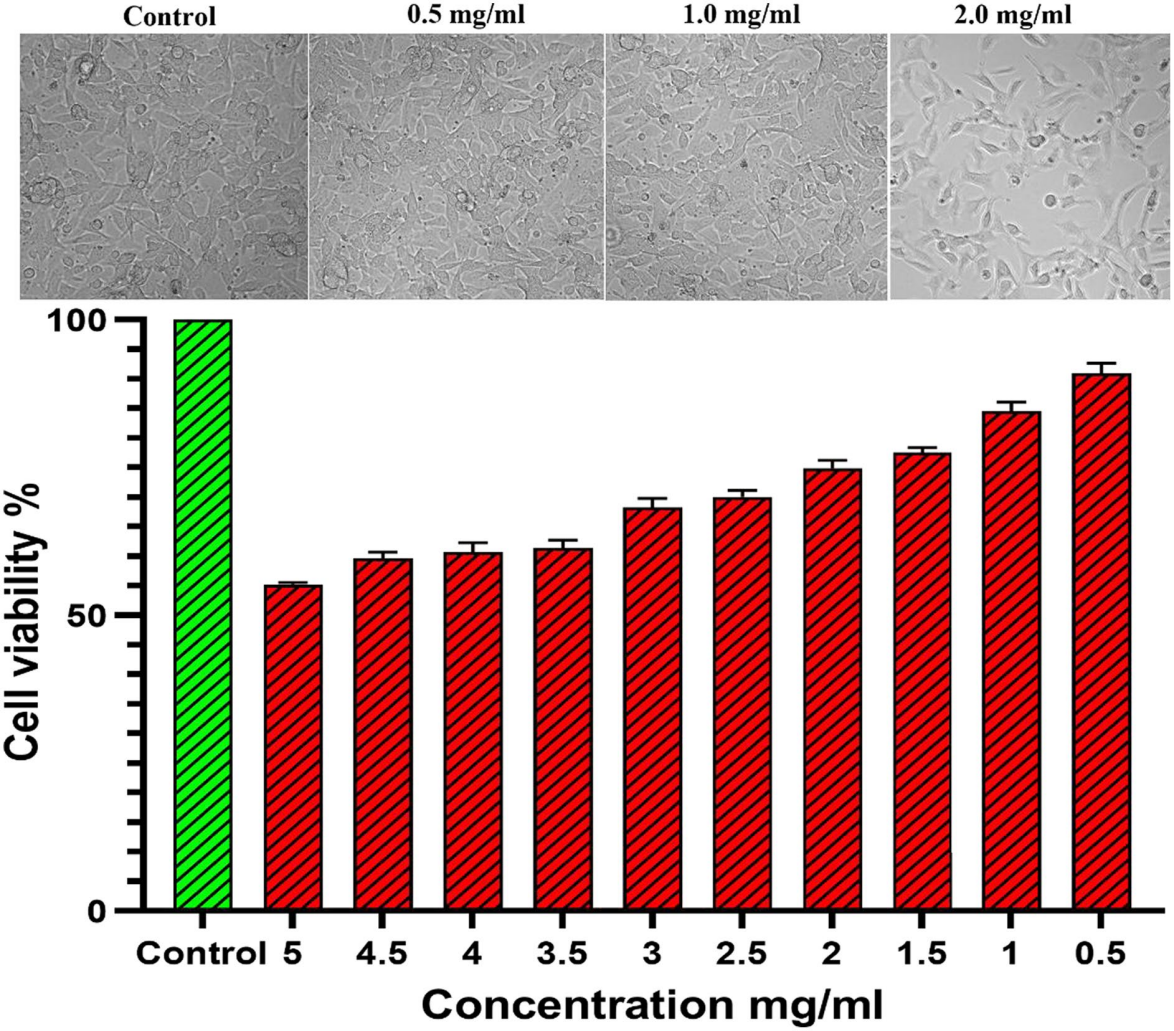
Cytotoxicity assay showing the cell viability in different dosage of CFEA.

The CAE-EI experiment did not show any significant changes when using a 3 mg concentration of plant extract dissolved in 0.5% DMSO as shown in Figure 8. For comparison, positive and negative controls of 0.1 N NaOH and 0.9% NaCl were used. The 0.1 N NaOH caused hemorrhaging at 0.5 min, vascular coagulation and lysis at 2 min, and worsening symptoms by 5 min. In contrast, neither 0.9% NaCl nor the CFEA showed noticeable changes. The irritation score (14.05) and severity score (3) contrasted with the value 0 of 0.9% NaCl revealed their strong irritating nature. The MTT test showed that solvent-free CFEA did not harm the metabolic activity of SIRC cells, indicating that the extracts are not toxic. A cytotoxicity test on SIRC cell lines showed that cell viability ranged from 90 to 55% at 0.5 to 5.0 mg/mL (Figure 9). According to current research, CFEA is a promising candidate for the development of a new topical antifungal agent because of its ability to inhibit biofilm-forming fungi, corneal permeability, pharmacodynamic properties, non-toxicity, and safety for corneal cells like SIRC cells.

### 3.7 Proposed synergistic mechanism of action CFEA phytocompounds

**Figure 10:**
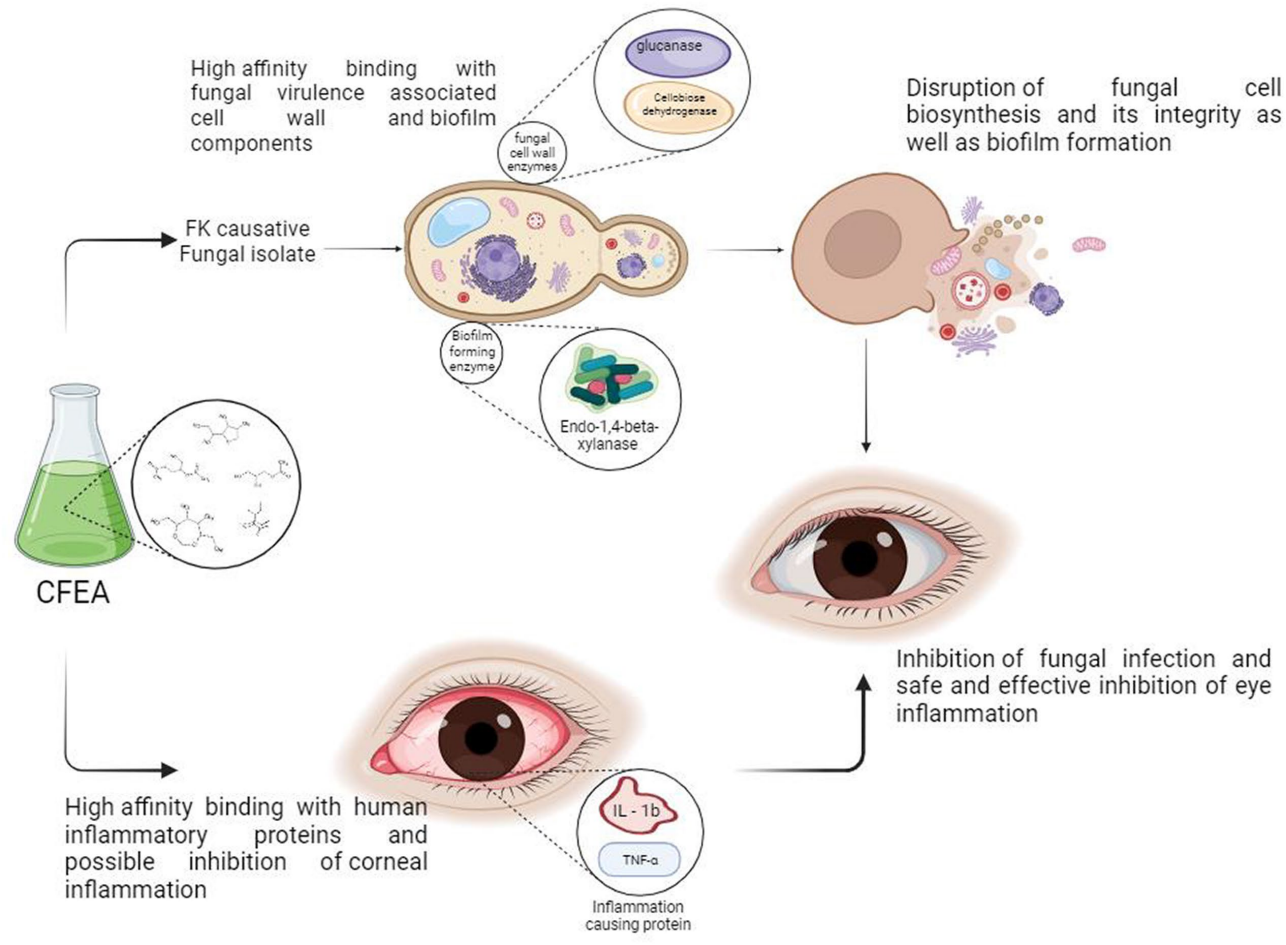
Proposed anti-keratitis mechanism of CFEA; credits to BioRender.com.

The antioxidant, antifungal, and antibiofilm properties of CFEA, along with the abundance of identified bioactive phytocompounds and their high-affinity interaction with fungal virulence target enzymes and human inflammatory proteins, have led us to propose the following mechanism of action for CFEA:

Figure 10 depicts the CFEA phytocomponents’ proposed general mechanism, qualifying them as constituents in a topical treatment to be produced to combat FK. When rich CFEA phytocompounds come into contact with the surface of the fungal cell wall, their interaction may cause a variety of damaging events at the cell wall, resulting in cell integrity breakdown and the release of internal components. They also inhibit biosynthesis as well as the development of new cell walls and biofilm. Furthermore, their in-silico druggable corneal permeability and other pharmacological features, as well as their high-affinity association with pathogenic and anti-inflammatory-linked proteins, may help to minimize post-infection inflammation in the eye.

## 4 Discussion

This study could significantly impact developing nations with agroeconomic sectors affected by FK. According to Niu et al. (2020), over a million new cases are documented annually. The study suggests that *C. ternatea* flower extract could be beneficial in inhibiting the growth and formation of biofilms by the FK-causing *C. hoffmannii* fungus, which are crucial components for corneal infection. The current work highlights how CFEA extract could be applied for the hard-to-manage FK biofilm formation and its control, which is important since biofilms once formed become extremely resistant to antibiotics (Roscetto et al., 2020). Overall, the study strongly recommends the natural remedies application for FK treatment and prevention. The outcomes of the current study corroborate with earlier studies which revealed that traditional eye disease remedies were shown to possess strong antifungal, antibiofilm, and antioxidant properties. According to Jeyaraj et al. (2022b), *C. ternatea*’s flower extract exhibits potent antibacterial and antifungal properties, even against microorganisms resistant to drugs. Jeyaraj et al. (2021) found that the methanolic extract of *C. ternatea* flowers exhibited MIC and MFC values of 0.8 and 1.6 mg/mL, respectively, against *Penicillium* and *Rhizopus* species. More potent antifungal activities against *Candida, Fusarium*, and *Aspergillus* species were found in methanolic and ethanolic extracts of *C. ternatea* seeds than in non-polar solvent extracts, according to research by Mushtaq et al. (2021). With MIC and MFC values of 0.5 and 1.0 mg, the CFEA extract exhibited the highest inhibition against *C. hoffmannii*.

The GCMS analysis revealed that the CFEA contains numerous phytochemicals with beneficial biological effects. Some of these substances have been identified as having antimicrobial, antioxidant, and anti-inflammatory properties, including hexanoic acid, 2-ethyl anhydride, n-Hexadecanoic acid (Thakur et al., 2018), glycerol 1,2-diacetate, Hexadecanoic acid (El-Sayed et al., 2023), 1,2,3-Propanetriol, 1-acetate, benzofuran, 2,3-dihydro- (Rani et al., 2023), 3-hydroxy-methyl ester (Kumar et al., 2021), isopropyl hexacosyl ether, and 5-Hydroxymethyl-2-furaldehyde (Fitriaturosidah et al., 2022). Additionally, phenol, 2,4-bis (1,1-dimethyl ethyl)-, has been found to have antifungal and biofilm-rupturing capabilities (Sharaf, 2020), while D-Glucitol, 1,4-anhydro-, has antiophidic effects (Singh et al., 2018). Among the 23 compounds discovered through GC–MS analysis, potentially druggable molecules having corneally permeable properties, which could be used to develop topical FK treatments, were screened, and their interaction with fungal target interaction is studied with *in silico* analysis.

According to Karami et al. (2022), a drug designed for corneal application should be a small, lipophilic molecule with minimal hydrogen bonds, in line with the composition of the corneal layers.

In our study, the compounds such as 9,9-dimethoxybicyclo[3.3.1]nonane-2,4-dione-; 2,5-O Methylene-D-mannitol; Glycerol 1,2 diacetate; D-Glucitol, 1,4-anhydro; and 1,2,3-Propanetriol exhibited favorable characteristics like good corneal permeability, low molecular weight, and lipophilic qualities. According to Khanzada et al. (2021), there is a significant association between the antifungal phytochemicals of a plant and the target elements of fungal virulence. These authors emphasized the importance of identifying molecular targets and understanding their mode of antifungal activity, whether it involves competitive or allosteric inhibition, to develop innovative antifungal therapy strategies.

Niu et al. (2020) found that the fungus in FK initially attaches itself to the corneal cell wall, leading to inflammation and redness in the cornea. Arana et al. (2009) also noted that the extracellular elements of the fungal cell wall play a crucial role in infiltrating host cells. Specifically, the hydrolytic enzymes in the fungal cell wall contribute to the deterioration of the corneal layers and the development of pathogenicity (Challacombe et al., 2019). Molecular docking receptors such as cellobiose dehydrogenase, Endo β 1,4 xylanase, and glucanase found in *C. hoffmanii*’s cell wall are involved in this process. Additionally, Endo β 1,4 xylanase has been recognized for its aggressive properties towards other fungi such as *Botrytis cinerea* and *Sclerotinia sclerotiorum* (Yu et al., 2016). Most antifungal therapeutics function by damaging the fungal cell wall, causing the contents to leak out and leading to fungal death (Hasim and Coleman, 2019). Several phytochemicals from *C. ternatea*, such as 9,9-dimethoxybicyclo[3.3.1]nonane-2,4-dione and 2,5-O Methylene-D-mannitol, have been found to have high binding affinity, similar to fluconazole. These compounds also exhibit strong receptor and ligand binding with the lowest negative MMGBSA score. Rolta et al. (2022) reported the highest binding score interaction between fungal pathogens and 9,9-Dimethoxybicyclo[3.3.1]nona-2,4-dione. According to Ololade Zacchaeus et al. (2021), these chemicals isolated from black velvet Tamarind seed extract have therapeutic benefits, including treating eye conditions. Additionally, the 9,9-Dimethoxybicyclo[3.3.1]nona-2,4-dione molecule is non-irritating to the eyes and has anti-inflammatory qualities (Reza et al., 2023). The stability of the complex is demonstrated by the MD simulation at 150 ns. In Figure 7A, the receptor and ligand complex reached equilibrium between 30 and 125 nanoseconds, showcasing its robust and stable nature. Similarly, in Figure 7B, the complex reached equilibrium between 40 and 95 ns. The slight variation in the RMSD was attributed to the flexibility of the ligand. Despite this volatility, the strong binding between the receptor and ligand indicates that the complex is stable.

This study also emphasized the importance of the anti-inflammatory properties of the CFEA, thereby reducing corneal inflammation. The high-affinity interactions between *C. ternatea* bioactive phytochemicals and human corneal inflammation-associated protein targets indicate their post-infection corneal inflammatory ailment-reducing potential. Recently number of studies (Iamsaard et al., 2014; GOH et al., 2022; Jeyaraj et al., 2022a) revealed the *C. ternatea* floral extract’s potentanti-inflammation, anti-proliferation, and oxidative stress reduction like protective functions. Their different biologically active compounds may be credited with these beneficial activities. In line with the above (Srichaikul, 2018) also demonstrated the absence of adverse reactions in rats receiving 2000 mg/kg *C. ternatea* flower ethanol extract. Rollando et al. (2023) also confirmed the CFEA safety as a pharmaceutical component with a high selectivity index value. The current study also shows that even at 3 mg/mL exposure, the extract did not harm SIRC cells since more than 70% of cells are viable. Hence, the CFEA is classified as a non-irritating one following the definition of Stanciauskaite et al. (2021). The CAE-EI assay used in this investigation also provided additional evidence of the extract’s anti-irritant properties.

## 5 Conclusion

The study highlights the potential of CFEA in combating biofilm-mediated fungal infections and inflammatory diseases. It suggests that the phytocomponents of CFEA offer safe and effective synthetic alternatives for FK-like eye disorders. CFEA contains numerous phytocompounds with important multipotent qualities such as antioxidant, antifungal, and antibiofilm properties. The study also identifies low-toxic, non-irritating, corneal permeable phytochemicals in CFEA that interact well with human inflammatory proteins and fungus-induced enzymes. As a result, the development of topical FK medicines has significantly progressed, and the traditional use of *C. ternatea* flower extract as a natural antifungal agent has been confirmed. Further research into the phytocompounds of CFEA may lead to safe and efficient FK treatments that can prevent biofilm formation.

## Data availability statement

The original contributions presented in the study are included in the article/supplementary material, further inquiries can be directed to the corresponding author.

## Author contributions

PY: Writing – original draft, Writing – review & editing. PJ: Methodology, Writing – review & editing. AJ: Visualization, Writing – review & editing. AS: Formal analysis, Writing – review & editing. PK: Review & editing. GP: Analysis and interpretation of results. AS: Review & writing. KM: Administration, Supervision, Writing & reviewing.

## Footnotes

^1^ https://www.uniprot.org

^2^ https://www.rcsb.org

^3^ https://pubchem.ncbi.nlm.nih.gov/
